# Facilely Synthesized Rough Photothermal Gold Nanoparticles for Portable and Rapid Detection of *Staphylococcus aureus*

**DOI:** 10.3390/bios16080415

**Published:** 2026-08-01

**Authors:** Zuoping Lv, Xiaoru Tan, Peilong Chen, Mingshuo Qu, Weijin Guo, Jihong Huang, Xin Cui

**Affiliations:** 1 Collaborative Innovation Center of Functional Food by Green Manufacturing, Food and Pharmacy College, Xuchang University, Xuchang 461000, China; lv2001@stu2024.jnu.edu.cn; 2 Key Laboratory of Biomaterials of Guangdong Higher Education Institutes, Department of Biomedical Engineering, Jinan University, Guangzhou 510632, China; txr2023@stu.jnu.edu.cn (X.T.); cpl3412@stu2024.jnu.edu.cn (P.C.); qms0108@stu2024.jnu.edu.cn (M.Q.); 3 Department of Biomedical Engineering, Shantou University, Shantou 515063, China; guoweijin@stu.edu.cn

**Keywords:** rough gold nanoparticles, photothermal biosensor, *S. aureus* detection, aptamer-based biosensing

## Abstract

Foodborne infections and enterotoxin poisoning caused by *S. aureus* pose serious threats to food safety and public health. However, conventional detection methods are often time-consuming, technically complex, and typically require relatively large sample volumes. Herein, we report an aptamer-functionalized rough gold nanoparticle (RPG)-based photothermal platform that enables rapid and quantitative detection of *S. aureus* using a smartphone-compatible thermal imaging system in combination with a laser and an infrared thermal camera. RPGs were synthesized via a facile one-step method, and exhibited uniform particle size and a measured photothermal conversion efficiency of ~73.20%. After optimization of the aptamer concentration, laser irradiation time, and probe concentration, the proposed method exhibited a wide detection range from 1.0 × 10^2^ to 1.0 × 10^7^ cfu/mL, with an operational detection threshold of 100 CFU/mL under the optimized experimental conditions, a total assay time of less than 40 min, and a sample requirement of only 30 μL per assay, while maintaining acceptable reproducibility. Spike-and-recovery experiments in real samples, including orange juice, tap water, and milk, yielded recoveries of 91.8–105.5%, indicating suitable analytical performance in complex matrices. This work provides a rapid, portable, and low-sample-volume detection strategy for *S. aureus*, offering a practical approach for on-site food safety monitoring.

## 1. Introduction

Rapid and on-site detection of foodborne pathogens is critically important for preventing food contamination incidents and mitigating public health risks [1]. *Staphylococcus aureus* (*S. aureus*) is one of the most frequently encountered bacterial contaminants in food products and processing environments [2,3]. Certain strains can produce heat-stable enterotoxins that remain active even after conventional thermal treatments, posing persistent safety concerns [4,5]. Notably, trace-level bacterial contamination may lead to toxin accumulation prior to sensory spoilage, underscoring the need for analytical platforms capable of sensitive and early-stage pathogen detection.

Conventional methods for *S. aureus* identification, including culture-based assays [4], polymerase chain reaction (PCR) [6], enzyme-linked immunosorbent assays (ELISAs) [7], and immunochromatographic tests, have been widely implemented in routine analysis. However, culture-based methods are inherently time-consuming, whereas PCR and ELISAs typically require sophisticated instrumentation, trained personnel, and multistep workflows [8]. Rapid test strips offer user-friendly operation, but generally suffer from limited sensitivity and provide only qualitative or semiquantitative results [9]. Collectively, these limitations restrict timely decision-making and hinder the deployment of existing technologies in resource-limited or on-site testing scenarios. To overcome these challenges, diverse biosensing strategies have been developed, including fluorescence [10], colorimetric [11], electrochemical [12], and surface-enhanced Raman scattering (SERS)-based platforms [13]. Although these approaches can achieve improved analytical sensitivity, many still rely on complex signal amplification processes, bulky readout instruments, or labor-intensive sample pretreatment, which compromise portability and real-world practicality. In addition, dependence on large optical/electrical detection systems remains a major bottleneck for truly field-deployable bacterial sensing technologies [14].

Photothermal sensing has recently emerged as an attractive signal transduction strategy, as it converts optical excitation into temperature variation that can be directly read by compact infrared devices or smartphone-assisted thermal imaging systems [15,16]. Compared with conventional optical readout modes, photothermal signals are less susceptible to background interference and do not require sophisticated optical components, making them well suited for portable and on-site applications [17]. In food safety monitoring, photothermal sensing offers additional advantages such as intuitive visualization, robust signal stability, and simplified operation. Several photothermal-based detection systems for *S. aureus* have been reported in recent years [18,19]. For example, Pt@Au@Ab1 nanoparticles have been employed to catalyze TMB color development while simultaneously generating photothermal signals, enabling dual-mode colorimetric–photothermal detection over a concentration range of 10–10^5^ cfu/mL [18]. A CPF-CRISPR platform integrating colorimetric, photothermal, and fluorescence readouts has demonstrated high-sensitivity detection of methicillin-resistant *S. aureus* (MRSA), achieving a fluorescence detection limit as low as 10 cfu/mL [20]. In another study, a sandwich-structured system based on magnetic beads, target bacteria, and CuS nanoparticles exploited the photothermal conversion and peroxidase-like activity of CuS to generate dual thermal and colorimetric signals, enabling quantitative detection from 10 to 10^6^ cfu/mL with portable infrared thermal imaging readout [21]. These studies collectively highlight the potential of photothermal strategies for constructing sensitive, visualized, and portable pathogen detection platforms.

Gold nanoparticles (AuNPs) have attracted significant attention due to their localized surface plasmon resonance (LSPR) effect, excellent biocompatibility, and tunable optical and photothermal properties [15,22]. However, conventional smooth-surfaced AuNPs typically exhibit limited optical absorption bandwidth, relatively low photothermal conversion efficiency, and suboptimal structural stability, which restrict their effectiveness in high-performance photothermal sensing applications [23]. To address these limitations, structurally engineered gold nanomaterials with coral-like [24], dendritic [25], or hollow morphologies [26] have been developed to enhance light-harvesting capability through plasmonic coupling, multiple scattering, and localized electromagnetic field amplification [24,27]. For instance, coral-like hollow gold nanospheres have been reported to achieve photothermal conversion efficiencies as high as 79.75% [24]. Nevertheless, such nanostructures require lipid-based templates or multistep synthetic procedures, resulting in complex fabrication processes and limited reproducibility. Bimetallic [28] or multimetallic nanocomposites [29] can further enhance photothermal responses, but are typically constrained by increased material costs, compositional complexity, and scalability challenges. Moreover, many existing photothermal sensing platforms still rely on high-grade laser systems or expensive infrared imaging devices [24,30], which undermines their suitability for low-cost and field-based food safety monitoring. Therefore, developing photothermal nanomaterials that simultaneously offer high photothermal conversion efficiency, facile synthesis, and compatibility with portable readout systems remains a critical challenge in advancing practical photothermal biosensing technologies.

Previously, we developed a one-pot synthesize method for the spontaneous growth of three-dimensional gold microparticles with abundant hot spots for robust SERS enhancement and sensitive and specific IL-6 quantification [31], while the photothermal performance of the as-synthesized nanoparticles was not explored. In this work, we report a photothermal biosensing platform based on rough-surfaced gold nanoparticles (RPGs) for the sensitive detection of *S. aureus*. The uniquely engineered nanostructure exhibits enhanced light-harvesting capability and photothermal conversion efficiency, enabling efficient thermal signal generation upon target recognition. By integrating aptamer-mediated molecular specificity, membrane-assisted bacterial enrichment, and smartphone-compatible infrared thermal readout, the proposed strategy enables sensitive, quantitative, and instrument-minimized detection of *S. aureus* in complex food matrices, demonstrating strong potential for on-site food safety monitoring. The novelty of this work lies in the integration of: (i) a rough gold substrate possessing strong photothermal conversion capability, (ii) aptamer-mediated target recognition, and (iii) a simple temperature-based readout for rapid detection of selective *Staphylococcus aureus*. Unlike previous studies that primarily utilized hierarchical gold nanostructures for SERS analysis or gold materials for biomarker detection, the present platform employs aptamer-functionalized RPGs as a photothermal sensing interface for bacterial detection without requiring Raman instrumentation, fluorescent labels, enzymatic amplification, or complex signal-processing procedures.

## 2. Materials and Methods

### 2.1. Materials and Reagents

Chloroauric acid (HAuCl_4_), silver nitrate (AgNO_3_), and hydroquinone were purchased from Sigma-Aldrich, St. Louis, MO, USA. Polyvinylpyrrolidone (PVP, Mn ≈ 130 kDa) was obtained from Shanghai Aladdin Biochemical Technology Co., Ltd., Shanghai, China, and N-methyl-2-pyrrolidone (NMP) was supplied by Shanghai Macklin Biochemical Co., Ltd., Shanghai, China. All reagents were of analytical grade and used as received without further purification. Ultrapure water (18.2 MΩ·cm) was used throughout all experiments. The aptamers of SA were synthesized by Shanghai Sangon Biotech Co., Ltd. Shanghai, China with a sequence of 5′-SH-GCAATGGTACGGTACTTCCTCGGCACGTTCTCAGTAGCGCTCGCTGGTCATCCCACAGCTACGTCAAAAGTGCACGCTACTTTGCTAA-3′ and 5′-SH-GCAATGGTACGGTACTTCCTCGGCACGTTCTCAGTAGCGCTCGCTGGTCATCCCACAGCTACGTCAAAAGTGCACGCTACTTTGCTAA-TAMRA-3′. Scrambled aptamer was synthesized with a sequence of 5′-SH-GTCAGCGTACATCGACTGGTACGTCTAGCGATGCTACGTCAGTAGCTCGATCGTAGCTACTGACGTCA-3′.

### 2.2. Instruments

Nanomaterials were washed using a high-speed centrifuge (D1008E, SCILOGEX, Rocky Hill, CT, USA; 5000 rpm).UV-visible absorption spectra were recorded with a UV-2550 spectrophotometer (Shimadzu, Suzhou, China). Transmission electron microscopy (TEM) images were acquired using a Zeiss ULTRA 55 field-emission TEM (Zeiss, Oberkochen, BW, Germany). A continuous-wave laser with a central wavelength of 638 nm served as the excitation source in photothermal experiments. Real-time temperature evolution and thermal imaging were monitored using a smartphone-compatible infrared thermal camera (HIKMICRO, Hangzhou, Zhejiang, China). The hydrodynamic particle size of different samples was measured using a Zetasizer Nano ZS (Malvern Instruments, Malvern, Worcestershire, UK).

### 2.3. Synthesis of RPGs

RPGs were synthesized at ambient temperature following a one-pot reduction protocol. In a typical procedure, 4.5 mL of PVP solution (90 mmol/L) was placed in a 10 mL round-bottomed flask, followed by the sequential addition of 160 µL of hydroquinone solution (42 mmol/L) and 60 µL of AgNO_3_ solution (10 mmol/L). Subsequently, 100 µL of HAuCl_4_ solution (40 mmol/L) was added dropwise under gentle stirring. The reaction mixture rapidly turned from colorless to dark gray, indicating nanoparticle formation. Afterwards, the product was transferred into centrifuge tubes and washed three times with NMP by centrifugation at 5000 rpm for 5 min to remove unreacted precursors. The resulting precipitate was redispersed in ultrapure water for further characterization and usage.

### 2.4. Bacterial Culture

Specific *Staphylococcus aureus* (ATCC 25923), *Saccharomyces cerevisiae* (ATCC 28339), *Escherichia coli* (ATCC 25922), and *S. epidermidis* (ATCC 12228) strains were obtained from laboratory stocks. Bacteria were cultured in Luria–Bertani (LB) broth at 37 °C with shaking (200 rpm) for 24 h until reaching the logarithmic growth phase. The optical density (OD) of the bacterial suspension was measured at 450 nm using a microplate reader. An OD_450_ value of 0.1 corresponded to an approximate bacterial concentration of 1.0 × 10^7^ cfu/mL. Serial dilutions with sterile phosphate-buffered saline (PBS, pH 7.4) were prepared to obtain the working concentrations ranging from 1.0 × 10^1^ to 1.0 × 10^7^ cfu/mL.

### 2.5. Probe Preparation and Characterization

To prepare the detection probe, the RPG dispersion was functionalized with aptamers through a surface conjugation process to yield RPG@Apt constructs. Prior to conjugation, the thiol-modified aptamer (5 µM) was reduced with tris(2-carboxyethyl)phosphine (TCEP, 1 mM) for 30 min to activate the sulfhydryl groups. Subsequently, 500 µL of the activated aptamer solution was mixed with an equal volume of RPG suspension (25 mg/mL) and incubated at 37 °C for 12 h to allow stable Au–S bond formation. The resulting aptamer-functionalized nanoparticles were collected by centrifugation at 5000 rpm for 10 min and washed twice with deionized water to remove unbound aptamers. The extent of aptamer conjugation was evaluated by UV-vis spectroscopic analysis.

To verify the successful conjugation of aptamers onto RPGs, a 3′-TAMRA-labeled aptamer was employed for fluorescence characterization. The fluorescence emission of TAMRA was recorded with an excitation wavelength of 560 nm, and the characteristic emission at 580 nm was monitored to confirm the successful immobilization of the fluorescently labeled aptamer on the RPGs surface. The surface charge were assessed by zeta-potential measurements, while dynamic light scattering (DLS) was used to determine the hydrodynamic size before and after aptamer conjugation. In addition, high-resolution X-ray photoelectron spectroscopy (XPS) S 2p spectra were acquired to characterize the chemical state of sulfur and to provide evidence for the Au–S interaction between the thiolated aptamer and the RPGs.

### 2.6. Photothermal Detection

For bacterial detection, 30 µL of *S. aureus* suspension was mixed with 30 µL of RPGs@Apt or bare RPGs without aptamer solution and incubated for 30 min to facilitate specific aptamer–target binding. The resulting mixture was then drawn into a syringe and passed through a 450 nm-pore membrane filter. This step retained the RPG@Apt–bacteria complexes on the membrane while removing unbound probes with a size of ~206 nm, achieving effective enrichment of the target complexes. The membrane was transferred to a clean glass slide and subsequently exposed to a 638 nm laser (power density: 0.7 W/cm^2^) for 3 min. During irradiation, temperature on the membrane surface was continuously recorded using a smartphone-based thermal imaging camera, and the corresponding infrared thermographs were obtained in real time. The total assay period was ~40 min.

### 2.7. Detection of S. aureus in Real Samples

To assess the applicability of the proposed photothermal detection assay in practical matrices, tap water, orange juice, and milk were selected. All samples were first filtered through sterile 0.22 µm membrane filters to remove suspended particulates. *S. aureus* at low (1.0 × 10^2^ cfu/mL), medium (1.0 × 10^4^ cfu/mL), and high (1.0 × 10^7^ cfu/mL) concentrations was spiked into 30 µL aliquots of each real sample for recovery analysis. Quantitative determination of *S. aureus* concentration was then performed using the optimized photothermal detection protocol. Note that method validation was performed only with spiked samples.

### 2.8. Statistics

All experiments were repeated in at least three independent experiments. Error bars in plots represent standard errors. The *p*-values were determined by two groups of data using Student’s two-tailed, unpaired *t*-test.

## 3. Results

### 3.1. Construction of a Photothermal and Visual S. aureus Detection Platform Based on RPGs

**Figure 1:**
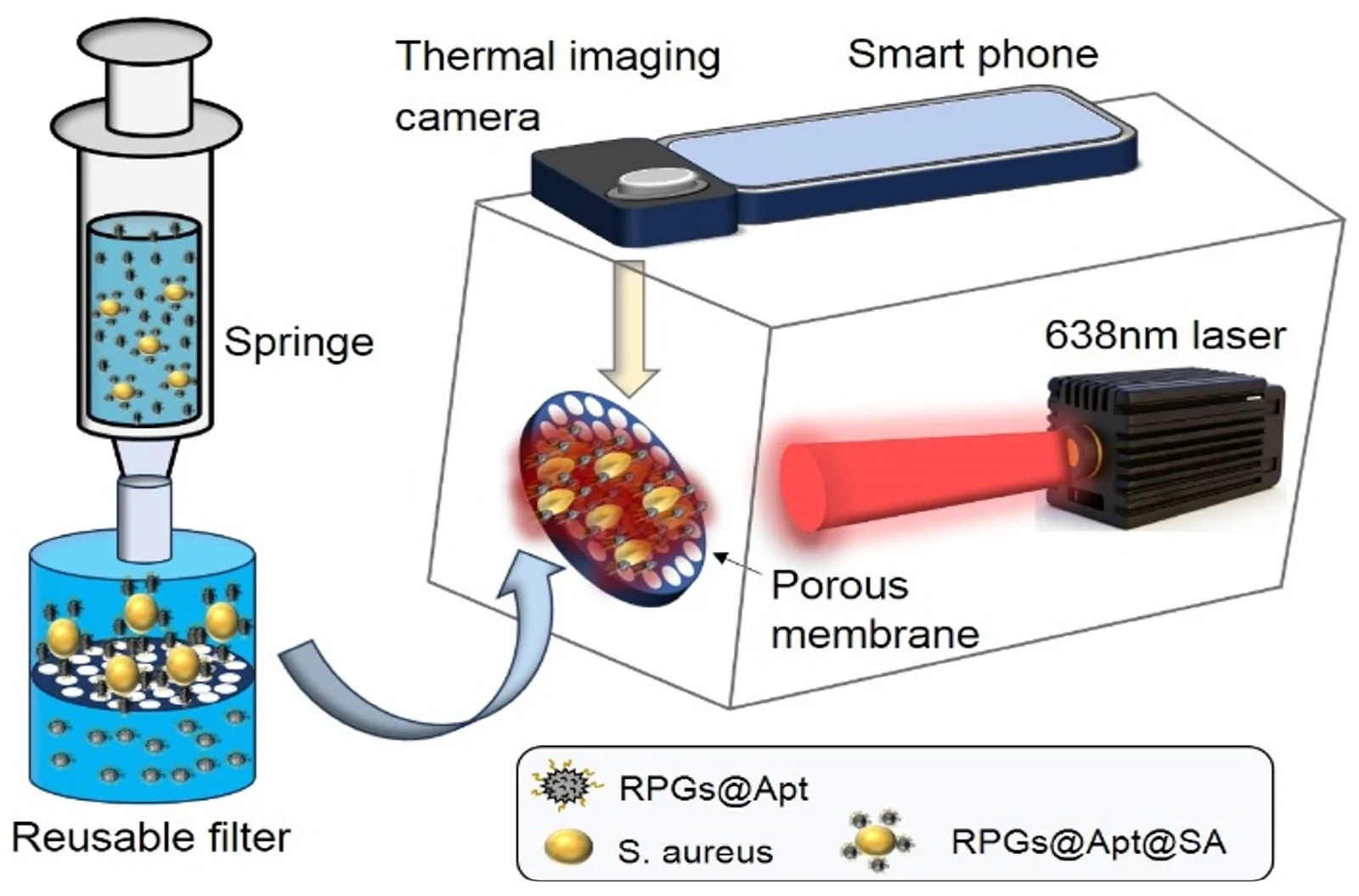
Schematic diagram of the photothermal detection platform for *S. aureus* based on RPGs.

Exploiting the distinctive structural features and potential photothermal properties of rough gold nanoparticles (RPGs) [13,31], we developed an aptamer-functionalized photothermal visualization platform for the quantitative detection of *S. aureus* (ATCC 25923). Briefly, the RPG@Apt probe was first prepared by incubating the RPGs with thiol-terminal aptamers for specific *S. aureus* recognition at an optimized ratio via the gold–sulfur bond [13]. Notably, the multifaceted prismatic protrusions and nanogap architectures of the RPGs provide numerous binding sites, enabling a favorable aptamer loading capacity and thereby enhancing the probe’s recognition efficiency toward the target bacteria. In the presence of the *S. aureus* target, the RPG@Apt probe specifically binds to the bacterial surface and form stable RPGs@Apt–bacteria complexes. To amplify the photothermal signal and minimize matrix interference, a membrane filtration–based enrichment strategy was employed with a membrane possessing a pore size of 450 nm. Owing to the significantly larger dimensions of RPGs@Apt–bacteria complexes (~1 µm) relative to the membrane pores, the RPGs@Apt–bacteria complexes were efficiently retained on the membrane surface, whereas unbound probes (~206 nm) were readily removed. This step enabled efficient enrichment and purification of the target complexes. Then, the membrane was retrieved, mounted onto a glass slide, and irradiated with a 638 nm laser, while real-time temperature variations at the membrane surface were simultaneously recorded using a smartphone-based thermal imaging camera. Finally, quantitative determination of *S. aureus* level was achieved by correlating the photothermal temperature increase with the bacterial concentrations (Figure 1).

### 3.2. Synthesis and Characterization of RPGs

**Figure 2:**
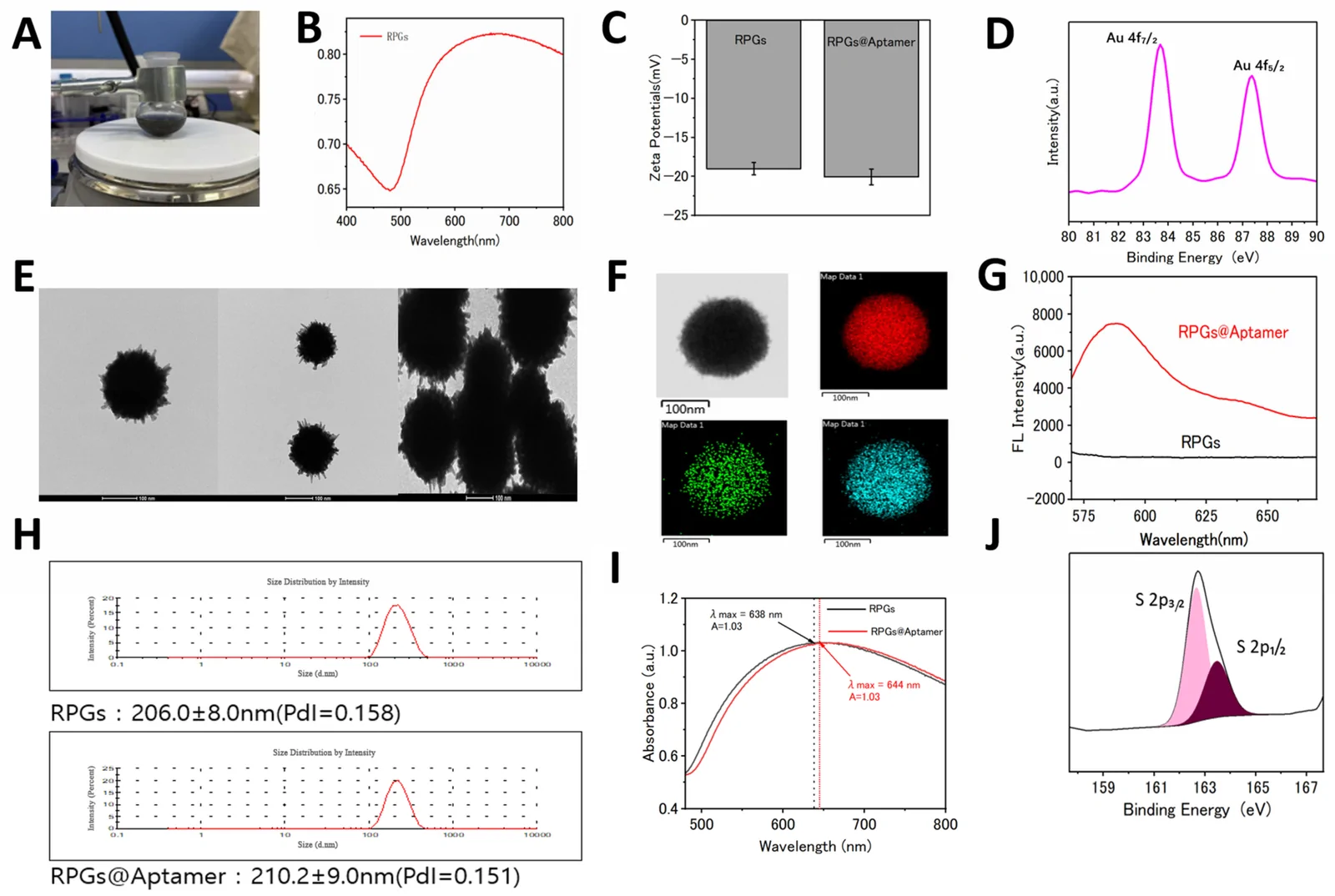
Preparation and characterization of RPGs and RPGs@Aptamer. (**A**) Photograph of RPG synthesis. (**B**) UV-vis absorption spectrum of RPGs. (**C**) Zeta potentials of RPGs and RPGs@Aptamer. (**D**) High-resolution XPS Au 4f spectrum of RPGs. (**E**) TEM images of RPGs. (**F**) TEM elemental mapping images of Au (red), Ag (green), and Cl (cyan) in RPGs. (**G**) Fluorescence spectra of RPGs and RPGs@Aptamer obtained using a TAMRA-labeled aptamer. (**H**) DLS hydrodynamic size distributions of RPGs and RPGs@Aptamer. (**I**) UV-vis LSPR absorption spectra of RPGs and RPGs@Aptamer. (**J**) High-resolution XPS S 2p spectrum of RPGs@Aptamer.

The RPGs were synthesized according to our previously reported one-step strategy with several modifications. As illustrated in Figure 2A, HAuCl_4_ was used as the gold precursor, hydroquinone served as the reducing agent, polyvinylpyrrolidone (PVP) acted as a stabilizing agent, and AgCl was introduced as a sacrificial template to regulate the formation of rough surface structures. After purification using N-methyl-2-pyrrolidone (NMP), the obtained RPGs were dispersed in ultrapure water for subsequent characterization. The successful formation of gold nanostructures was initially verified by UV-vis spectroscopy, where RPGs exhibited a characteristic localized surface plasmon resonance (LSPR) absorption band (Figure 2B).

The chemical state of gold was further analyzed by XPS. The high-resolution Au 4f spectrum showed two characteristic peaks at 83.68 eV and 87.38 eV, assigned to Au 4f_7_/_2_ and Au 4f_5_/_2_, respectively, indicating the presence of metallic Au^0^ in RPGs (Figure 2D) [32]. TEM images revealed that RPGs possessed irregular polyhedral structures with abundant surface protrusions and nanoscale rough features (Figure 2E). The elemental mapping results further demonstrated the uniform distribution of Au, Ag, and Cl elements throughout the nanoparticles, confirming the successful formation of RPGs (Figure 2F).

The construction of the RPG@Aptamer probe was subsequently verified by multiple characterization techniques. Using a TAMRA-labeled aptamer, RPGs@Aptamer exhibited a strong TAMRA fluorescence signal, whereas bare RPGs showed negligible fluorescence, confirming successful aptamer immobilization (Figure 2G) [33]. Zeta-potential analysis revealed a slight change in surface charge after aptamer modification, attributed to the introduction of negatively charged phosphate backbones from aptamers (Figure 2C) [34]. DLS measurements showed an increase in hydrodynamic diameter from 206 nm to 210 nm after aptamer conjugation, suggesting the formation of an aptamer coating layer (Figure 2H) [35]. Meanwhile, the LSPR peak shifted from 638 nm to 644 nm after modification, which was possibly attributable to the increased local refractive index caused by aptamer attachment (Figure 2I) [36]. Finally, XPS S 2p analysis was performed to investigate the surface chemical modification of RPGs@Aptamer. The S 2p spectrum was deconvoluted into two peaks at 162.7 eV (S 2p_3_/_2_) and 163.9 eV (S 2p_1_/_2_), corresponding to sulfur species from thiolated aptamers, providing evidence for Au–S interactions between aptamers and RPGs (Figure 2J) [37]. These results collectively demonstrated the successful preparation of RPGs and the effective construction of the RPG@Aptamer probe.

### 3.3. Evaluating the Photothermal Performance of RPGs

The photothermal performance of nanomaterials is closely correlated with their micro- and nanostructural characteristics [16,22,26]. Inspired by previous reports demonstrating that three-dimensional hierarchical plasmonic nanostructures enhance photothermal efficiency by improving light-harvesting capability [38], we systematically evaluated the photothermal properties of the synthesized RPGs. UV-vis absorption spectra showed that the RPGs exhibited strong and broadband optical absorption in the range of 400–800 nm, with a characteristic absorption peak at ~638 nm (Figure 2B). Accordingly, a 638 nm laser was selected as the excitation source in subsequent photothermal experiments to maximize photothermal conversion efficiency.

**Figure 3:**
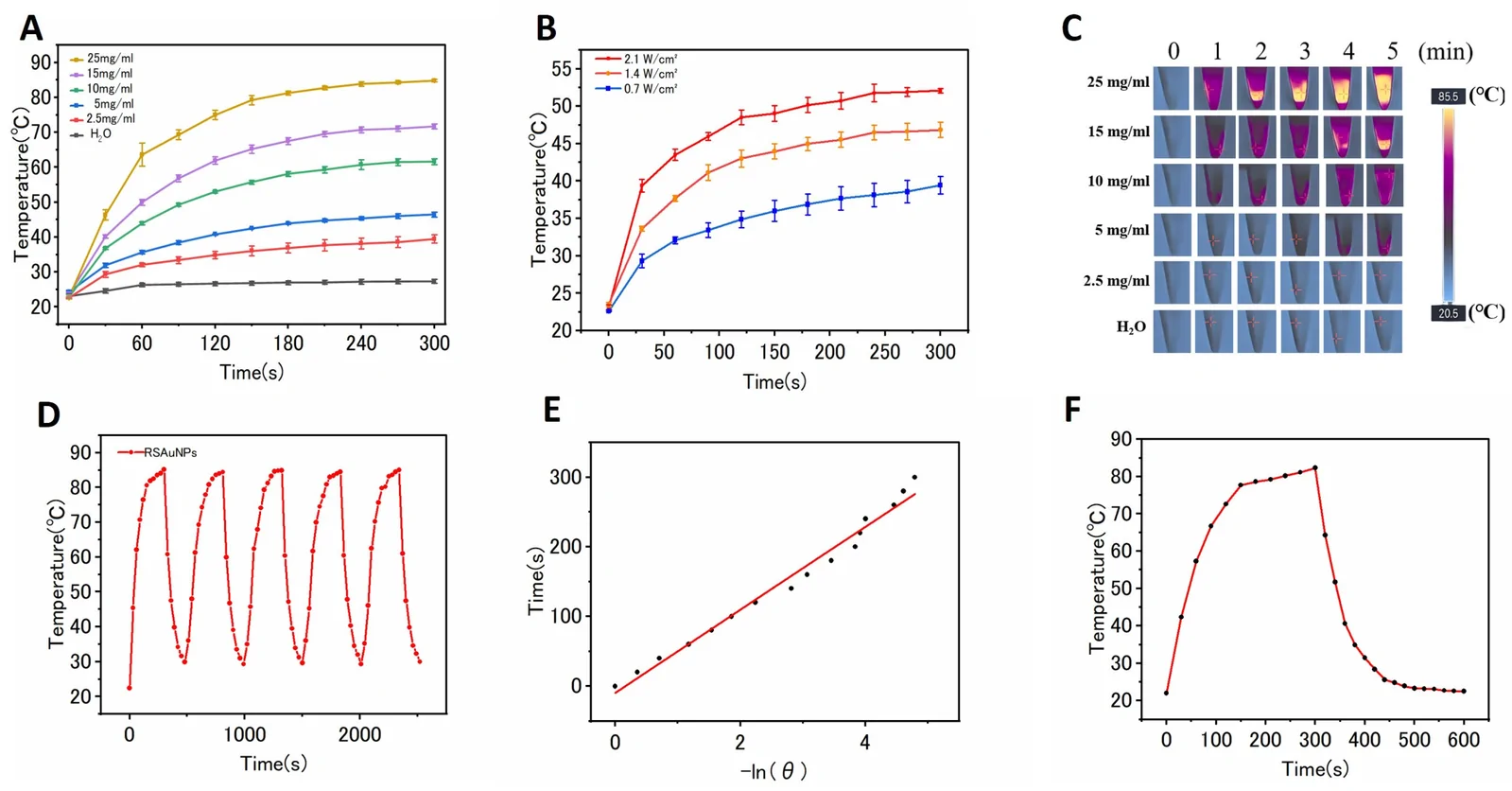
Photothermal performance characterization of RPGs. (**A**) Temperature variation in RPGs at different concentrations with laser irradiation time. (**B**) Temperature variation in RPGs under different laser power with irradiation time. (**C**) Photothermal images of water and RPGs at different concentrations under 638 nm laser irradiation. (**D**) Photothermal stability of RPGs after five on–off laser cycles. (**E**) Linear fitting plot of −ln(θ) versus time during the cooling process. (**F**) Temperature profile of one heating–cooling cycle.

We then explored the effects of both nanoparticle concentrations and laser intensity in regulating the photothermal performance (Figure 3). The laser power density was first fixed at 0.7 W/cm^2^, and RPGs with a concentration ranging from 2.5 to 25 mg/mL were irradiated (Figure 3A,C). Our results showed that both the heating rate and equilibrium temperature increased positively with the increasing concentrations of RPGs. Notably, the 25 mg/mL RPG solution induced a maximum temperature of 85.0 °C after 300 s of irradiation, whereas ultrapure water under identical conditions exhibited a negligible temperature increase of only 3.6 °C, demonstrating the distinct photothermal conversion capability of the RPGs (Figure 3C). In addition, the sensing configuration involves membrane-retained complexes rather than freely dispersed nanoparticles, and the presence of bacterial cells and aptamer layers may partially influence light absorption and heat transfer. Consequently, different equilibrium temperatures can be obtained even under the same laser power density. In addition, when the RPG concentration was fixed at 25 mg/mL, increasing the laser power density from 0.7 to 2.1 W/cm^2^ led to an increase in equilibrium temperature from 38.6 °C to 52.4 °C, indicating that the photothermal output can be efficiently tuned by adjusting the laser power to meet diverse application requirements (Figure 3B).

To investigate photothermal stability, we also irradiated the RPGs using five consecutive laser on–off cycles (Figure 3D). In each cycle, the RPGs were irradiated with a 638 nm laser at 0.7 W/cm^2^ for 300 s, followed by natural cooling to room temperature. The temperature–time profiles remained nearly identical over all five cycles, with the fluctuations in equilibrium temperature less than 2 °C, indicating that the RPGs remained intact without aggregation or structural degradation during repeated photothermal cycling. Hence, these results demonstrate acceptable structural stability and photothermal reproducibility of RPGs.

The photothermal conversion efficiency (η) of the RPGs was calculated using the time-constant method [39]. Briefly, the cooling curve was recorded after laser irradiation, and −ln(θ) was plotted against the cooling time, where θ = (T − T_0_)/(T_ax_ − T_0_), T_0_ is the ambient temperature, and T_ax_ is the maximum temperature. Linear fitting yielded the equation Y = 52.28x − 2.00 (R^2^ = 0.98) (Figure 3E,F). Under irradiation at 785 nm, the RPGs exhibited a PCE of ~73.20% (Table 1). The photothermal performance of the RPGs can be attributed to three synergistic mechanisms: (i) the multilevel surface protrusions and architecture induce multiple scattering and reflection of incident light, effectively extending the optical path length within the material and increasing the probability of photon absorption [40]; (ii) the framework and surface asperities generate localized electromagnetic field enhancement, thereby intensifying LSPR effects and accelerating the conversion of photon energy into thermal energy [41]; and (iii) the large specific surface area not only improves light-harvesting efficiency but also facilitates heat transfer from the nanoparticles to the surrounding medium, enabling fast and efficient temperature elevation [42].

### 3.4. Optimization of Experimental Parameters

**Figure 4:**
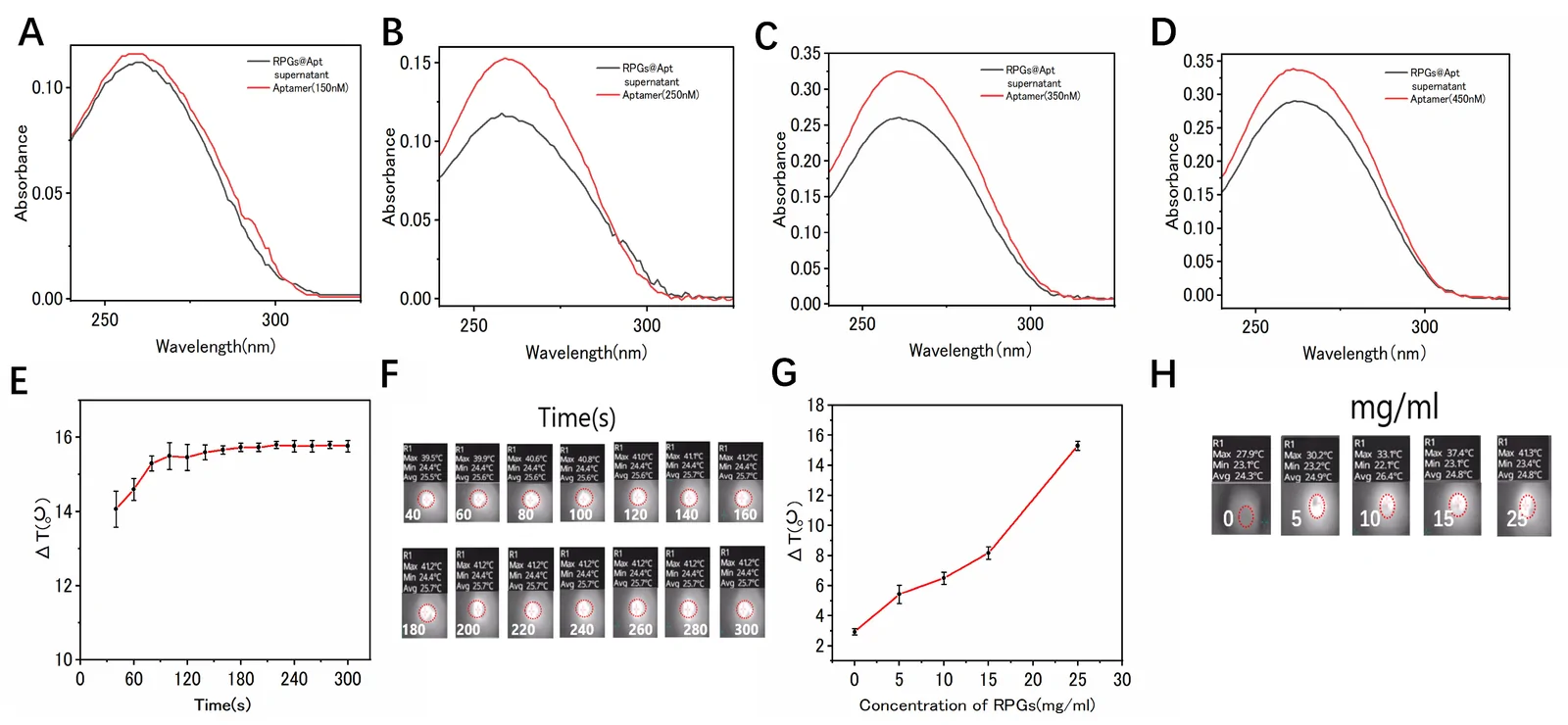
Parameter optimization. (**A**–**D**) Absorption spectra of aptamer solutions at 150 nM, 250 nM, 350 nM, and 450 nM and the supernatants obtained after incubation of aptamer solutions with RPGs particles followed by centrifugation. (**E**,**F**) Optimization of laser irradiation time and corresponding images from the imager software. (**G**,**H**) Optimization of RPG particle concentration and the corresponding images from the imager software.

To achieve optimal analytical performance, key parameters, including aptamer density, laser irradiation period, and concentration of the RPG@Apt probe, were systematically investigated. To quantify the level of aptamer conjugated on RPGs, aptamer solutions with concentrations of 150, 250, 350, and 450 nM were conjugated with the RPGs, and the UV absorbance of the resulting supernatants at 260 nm was measured (Figure 4A–D). The absorbance difference at 260 nm (Δ260), defined as the difference between the initial aptamer solution and the post-modification supernatant, was employed as an indicator of aptamer conjugation efficiency. As the aptamer concentration increased from 150 to 350 nM, Δ260 values increased progressively, indicating enhanced immobilization efficiency. However, further increasing the concentration beyond 350 nM resulted in a plateau in Δ260, likely due to the surface-crowding effects that may hinder aptamer attachment [48]. Accordingly, 350 nM was identified as the optimal aptamer concentration.

Laser irradiation time is another critical factor governing both signal stability and detection efficiency (Figure 4E,F). Duration of laser exposure was regulated over a range of 40–300 s, and the corresponding temperature increase was monitored in real time. During the initial irradiation phase (40–180 s), the temperature increased rapidly, attributable to the efficient photothermal conversion of the RPGs. Beyond 180 s, the rate of temperature increase markedly decreased and gradually reached a steady state, indicating that thermal equilibrium between heat generation and dissipation had been established. Prolonged irradiation beyond this point did not yield additional signal enhancement and may further compromise reproducibility due to excessive heating. Therefore, an irradiation time of 180 s was selected as the optimal condition, balancing signal robustness and operational efficiency.

We further optimized the concentration of RPGs@Apt applied for *S. aureus* detection (Figure 4F,H). The results demonstrated that ΔT (defined as the temperature difference between positive and negative samples) increased substantially with the concentration of RPGs when below 25 mg/mL. This trend can be attributed to the increased number of RPGs per unit area, leading to higher cumulative heat generation upon laser irradiation. Beyond the concentration of 25 mg/mL, ΔT reached a maximum value of 16.8 °C. Thus, 25 mg/mL was selected for subsequent experiments.

### 3.5. Detection Sensitivity

**Figure 5:**
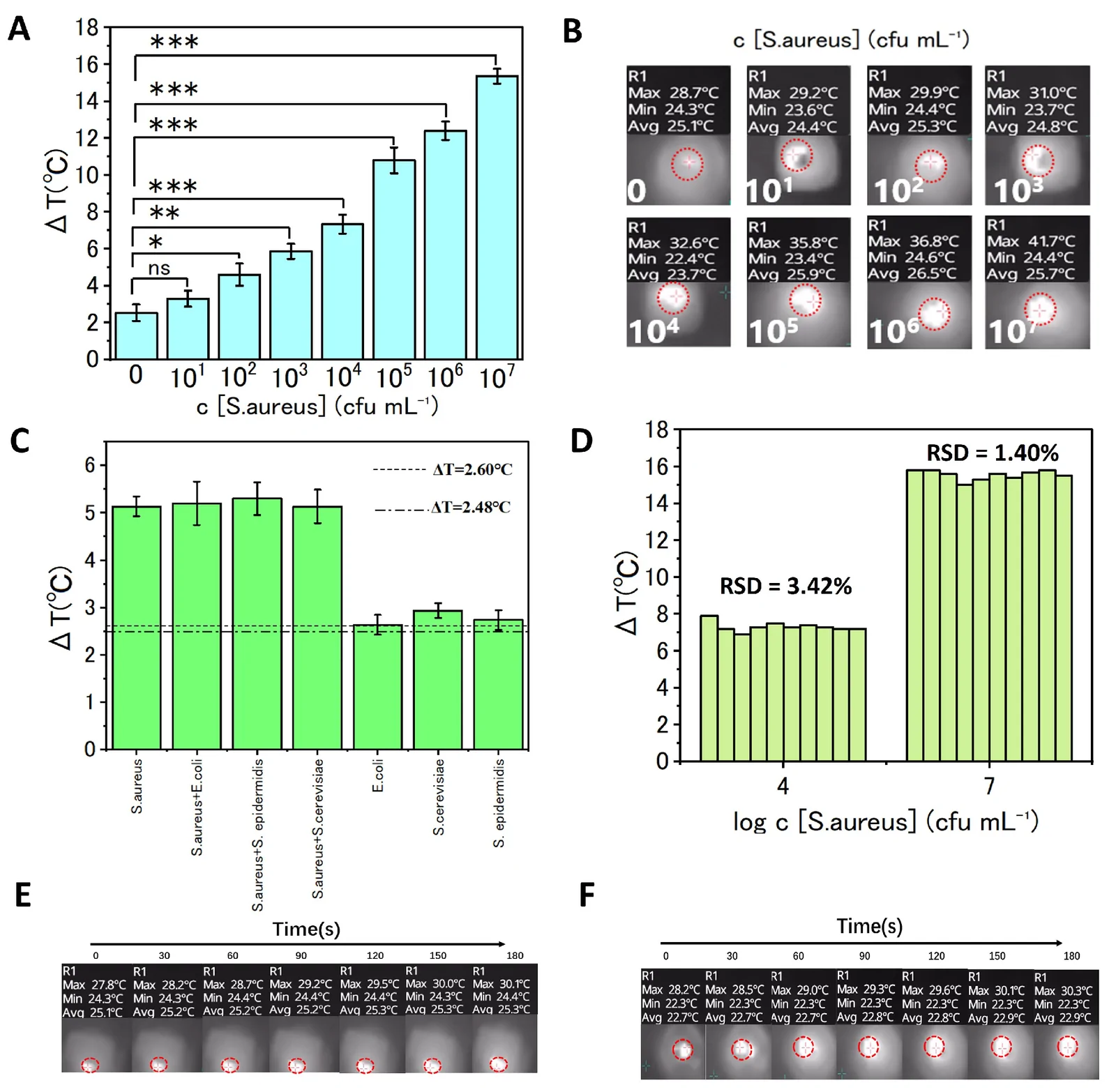
Analytical performance of RPGs for *S. aureus* detection. (**A**) Photothermal ΔT responses to serial concentrations of *S. aureus* (ATCC 25923) ranging from 0 to 10^7^ cfu/mL (ns: *p* > 0.05; *: *p* < 0.05; **: *p* < 0.01; ***: *p* < 0.001). (**B**) Corresponding thermal imaging photographs recorded by infrared imager software under each bacterial concentration. (**C**) *S. aureus* (ATCC 25923) detection selectivity analysis. Note that the ΔT = 2.60 °C was for bare RPGs (without aptamer functionalization) and ΔT = 2.48 °C was for aptamer-conjugated RPGs with a bacteria concentration of 0 cfu/mL. (**D**) Inter-batch stability analysis. (**E**) Representative time-lapsed photothermal images of RPGs@Apt incubated with *S. epidermidis*. (**F**) Representative time-lapsed photothermal images of RPGs@Apt incubated with scrambled aptamer-modified RPGs.

Under the optimized experimental conditions, the analytical sensitivity of the proposed photothermal detection platform was evaluated (Figure 5). A standard strain of *S. aureus* with concentrations ranging from 1.0 × 10^1^ to 1.0 × 10^7^ cfu/mL was employed as the target bacterium. After incubation the bacterial dilution with the RPG@Apt probes, the solution was filtered using a porous membrane, and the retained complexes on the membrane were subsequently irradiated with a 638 nm laser at a power density of 0.7 W/cm^2^ for 180 s (Figure 5B). Temperature changes on the membrane surface were recorded using a portable smartphone-based thermal imaging system. Under optimized experimental parameters, we systematically characterized the photothermal signaling performance of the proposed biosensor toward serially diluted *Staphylococcus aureus* suspensions (Figure 5A). The horizontal axis corresponds to the blank control (0 cfu/mL) and gradient bacterial concentrations ranging from 10^1^ to 10^7^ cfu/mL, while the vertical axis records the corresponding photothermal temperature shift ΔT, with error bars denoting standard errors of three independent experimental replicates. The blank group merely produced negligible baseline thermal signals. At 10^1^ cfu/mL, the measured ΔT exhibited no statistically significant difference compared with blank samples (*p* > 0.05), which failed to separate target bacteria from blank matrix effectively. When the bacterial concentration reached 100 cfu/mL, distinct photothermal responses were obtained with statistical significance (*p* < 0.05), and the statistical discrepancy was further strengthened at higher concentrations (*p* < 0.01; *p* < 0.001). Overall, ΔT increased monotonically along with ascending bacterial loads and reached a maximum signal at 10^7^ cfu/mL. According to statistical comparison between each test group and blank control, the operational limit of detection (LOD) of this sensing platform was defined as 100 cfu/mL (Table 2).

### 3.6. Detection Selectivity and Stability

To assess the selectivity of the developed RPG-based photothermal assay for *S. aureus* detection, several different bacteria frequently encountered in food and environmental samples were selected, including *Escherichia coli*, *Saccharomyces cerevisiae* and *S. epidermidis* with a low concentration of 1.0 × 10^2^ CFU/mL (Figure 5C). The results revealed that *S. aureus* (ATCC 25923) produced a clear temperature increase (~5.2 °C), whereas all non-target bacteria exhibited significantly lower ΔT values (<3 °C), comparable to that of the blank control. Note that the photothermal response for *S. aureus* at 10^2^ CFU/mL was consistent with the sensitivity evaluation, in which this concentration was identified as the lowest concentration producing a statistically significant increase compared with the blank control (*p* < 0.05). Furthermore, the corresponding time-lapsed thermal images showed relatively weak temperature responses (Figure 5E), which were significantly lower than that obtained for *S. aureus* (ATCC 25923). These findings demonstrate the selective response of the detection platform toward *S. aureus* (ATCC 25923), likely involving aptamer-mediated recognition, although contributions from nonspecific retention cannot be excluded. It should be noted that aptamer-mediated bacterial recognition can be influenced by strain-specific surface characteristics. Therefore, the selectivity demonstrated in this study represents recognition of the tested *S. aureus* strain and should not be interpreted as universal recognition of all possible *S. aureus* isolates. Moreover, as the negative charge of the synthesized RPGs, the RPGs without aptamer may have limited interference with biosensor performance. To verify that, bare RPGs (without aptamer functionalization) were incubated under identical conditions and tested with a bacterial concentration of 1.0 × 10^2^ CFU/mL. The resulting temperature change (*n* = 3) was ~2.60 ± 0.50 °C and matched the baseline response in buffer (~2.48 ± 0.46 °C), as shown in Figure 5C. Furthermore, to investigate whether nonspecific bacterial retention could occur at a much higher bacterial concentration, an additional control experiment was performed using bare RPGs incubated with 1.0 × 10^7^ CFU/mL of *S. aureus*. Even under this high bacterial loading condition (Figure S1), bare RPGs did not produce a significant increase (~2.71 ± 0.53 °C) in photothermal response, demonstrating that the enhanced signal generated by aptamer-functionalized RPGs mainly resulted from specific recognition between the aptamer and target bacteria. Scrambled aptamer-modified RPGs were incubated with *S. aureus* under identical conditions (Figure 5F), with a resulting temperature change (*n* = 3) of ~2.54 ± 0.41 °C. The negligible variation in photothermal signals among these control groups demonstrated that the signal enhancement was mainly attributed to the specific interaction between the target-specific aptamer and *S. aureus*, rather than nonspecific trapping or random adsorption.

To explore the practical application potential of the proposed detection strategy, its stability was further evaluated via inter-batch reproducibility tests. Under identical experimental conditions, ten independently synthesized batches of RPG probes (*n* = 10) were employed for quantitative detection of *S. aureus* samples at concentrations of 1.0 × 10^4^ cfu/mL and 1.0 × 10^7^ cfu/mL. The corresponding inter-batch relative standard deviations (RSDs) for the two concentration levels were 3.42% and 1.40% (Figure 5D), respectively, demonstrating the outstanding detection consistency of this sensing system among different preparation batches.

### 3.7. Application in Real Samples

To validate the applicability of the proposed platform in real-world scenarios, recovery experiments were conducted with three real matrices: tap water, orange juice, and milk. Their original pH values were 7.1 ± 0.2 (tap water) and 3.5 ± 0.3 (orange juice). All samples were pre-filtered with a 0.22 μm sterile filter without additional dilution, and a 30 μL filtrate was used for tests. The recovery rate was calculated as recovery (%) = (measured bacterial concentration/theoretical spiked bacterial concentration) × 100%, with five parallel samples for each group. The samples were spiked with low (1.0 × 10^2^ cfu/mL), medium (1.0 × 10^4^ cfu/mL), and high (1.0 × 10^7^ cfu/mL) concentrations of *S. aureus*. Each concentration was analyzed using the optimized detection protocol. The recovery rates for tap water samples ranged from 93.1% to 104.8% with RSDs of 3.0–4.5%, recovery rates of 95.2–105.5% with RSDs of 2.6–4.1% for orange juice samples, and recovery rates of 91.8–101.3% with RSDs of 3.3–4.9% for milk samples. Taken together, these results demonstrate that the platform effectively mitigates matrix interference and enables accurate detection of *S. aureus* in complex real samples.

## 4. Conclusions

In this work, photothermal rough gold nanoparticles (RPGs) were fabricated via a facile one-step route. Combined with *S. aureus*-specific aptamers, a proof-of-concept photothermal sensing platform was built for rapid, low-volume, visual quantitative detection of *S. aureus*. Under optimal conditions, it achieves a wide linear range of 1.0 × 10^2^–1.0 × 10^7^ cfu/mL and an operational detection limit of 100 cfu/mL under the optimized experimental conditions, with favorable storage stability and inter-batch reproducibility. Spike-recovery assays on orange juice, tap water, and milk produce recoveries of 91.8–105.5%. Quantitative analysis can be readily completed with a portable laser and smartphone-matched thermal imager. Future efforts will refine this system through full structural characterization of RPGs and tests on naturally contaminated samples and strain-dependent recognition limitation. This lightweight, low-consumption strategy addresses the drawbacks of time-consuming, instrument-reliant traditional pathogen detection. By enriching aptamer targets and miniaturizing the device, the platform holds great potential for multiplex on-site food safety screening.

## Figures and Tables

**Table 1:** Reported photothermal conversion efficiency (PCE) of RPGs and other representative photothermal materials.

	Particle Size (nm)	Irradiation Wavelength (nm)	Photothermal Conversion Efficiency (%)	Target	Ref.
TAT-Pd@Au/Ce6 MnO2 NPs	140	670	56.90	4T1 tumor-bearing mice	[43]
DPP-TPA	152	660	48.00	Tumor blood vessels	[44]
PPor-PEG NPs	81	630	62.30	A546 human lung adenocarcinoma cells	[45]
CuS@BSA	4	660	29.80	*S. aureus* and *E. coli*	[46]
Au NSs	29	785	34.50	A546 human lung adenocarcinoma cells	[47]
This work	206	638	73.20	*S. aureus*	

**Table 2:** Comparison of representative recently reported *S. aureus* detection methods.

Method/Sensor	Detection Principle	Linear Range	Limit of Detection (LOD)	Assay Time	Sample Volume	Analytical Matrix	Instrumentation Requirements	Practical Considerations	
Electrochemical Immunosensor	Protein A recognition electrochemistry	~10^2^–10^9^ CFU/mL	13–150 CFU/mL	20–60 min	40–50 μL	Milk, serum, PBS	Electrochemical workstation	Specific binding, vulnerable to contamination	[49]
Paper-based Electrochemical Sensor	Paper-based bacterial metabolic detection	~10^3^–10^5^ CFU/mL	~10^4^ CFU/mL	~1 h (real-time)	40 μL	Food complex matrices	Portable potentiostat, smartphone interface	Low-cost portable, non-strain-specific, matrix-prone	[50]
Flexible PDMS-AuNP SERS Chip	Flexible substrate SERS fingerprint detection	~10^2^–10^6^ CFU/mL	3.42 CFU/mL	~30 min	N/A	Clinical wound exudate	Confocal Raman spectrometer	High sensitivity, adaptable to irregular wounds, deformation interferes signal	[51]
Photothermal-SERS Lateral Flow Strip	Photothermal + SERS	~10^1^–10^8^ CFU/mL	10 CFU/mL	~80 min	~50 μL	Milk, biological fluids	Confocal Raman spectrometer (for SERS mode)	High sensitivity and easy operation, needs Raman device	[52]
Conventional qPCR	DNA amplification & SYBR Green fluorescence detection	~10^1^–10^8^ CFU/mL	~10^1^–10^2^ CFU/mL	2–4 h	5–50 μL	Pure cultures, extracted DNA from various samples	Real-time PCR thermal cycler	High-specificity gold standard, time-consuming, costly, and labor-intensive	[53]
Our work	Aptamer-based photothermal rapid detection	~10^2^–10^7^ CFU/mL	~100 CFU/mL	~40 min	30 μL	Complex biological samples	Infrared thermal camera or photothermal detection setup	Rapid, cost-effective, easy to operate, and applicable to a wide range of scenarios	

## Data Availability

The original contributions presented in this study are included in the article/Supplementary Material. Further inquiries can be directed to the corresponding author.

## Supplementary Materials

The following supporting information can be downloaded at https://www.mdpi.com/article/10.3390/bios16080415/s1. Figure S1: The representative time-lapsed photothermal images for bare RPGs with a bacterial concentration of 1.0 × 10^7^ cfu/mL.
