# Supplementary material for: Facilely Synthesized Rough Photothermal Gold Nanoparticles for Portable and Rapid Detection of Staphylococcus aureus
Source: Biosensors (Basel). 2026 Aug 1;16(8):415. doi: 10.3390/bios16080415 (PMC13510419; doi:10.3390/bios16080415)
Supplement: Supplementary file 1 [file biosensors-16-00415-s001.zip › biosensors-4356249-supplementary.pdf]

Supporting Information

Facilely Synthesized Rough Photothermal Gold Nanoparticles for Portable and Rapid Detection of Staphylococcus aureus

Zuoping Lv[1,2,4], Xiaoru Tan[2], Peilong Chen[2], Mingshuo Qu[2],Weijin Guo[3,4], Jihong Huang[1*]and Xin Cui[2*]

1 Collaborative Innovation Center of Functional Food by Green Manufacturing, Food and Pharmacy College, Xuchang University, Xuchang 461000, China

2 Key Laboratory of Biomaterials of Guangdong Higher Education Institutes, Department of Biomedical Engineering, Jinan University, Guangzhou 510632, China

3 Department of Biomedical Engineering, Shantou University, Shantou 515063, China

4 These authors contribute equally to this work: Zuoping Lv and Weijin Guo.

*Correspondence should be addressed to X. Cui (cx2019@jnu.edu.cn) and J.H. Huang (huangjih1216@126.com)

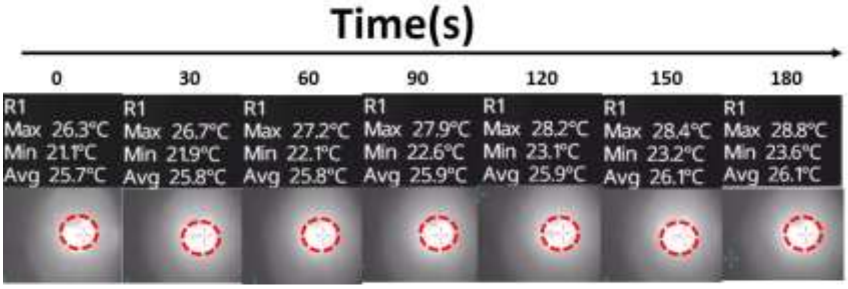

Figure s1. The representative time-lapsed photothermal images for bare RPGs with a bacterial concentration of 1.0×10^7 cfu/mL.
